# Bioactive Fiber and Polyphenols: Composition and Association with Fecal Lipid Profiles in Mango Bagasse and Peel Confectionery

**DOI:** 10.3390/ijms27031166

**Published:** 2026-01-23

**Authors:** Yuritzi Barbosa, Alejandro Castrejon, Marcela Gaytán-Martínez, Jimena Álvarez-Chávez, Adriana Chico-Peralta, Mar Villamiel, Marcelo Hernández-Salazar, Aurea K. Ramírez-Jiménez

**Affiliations:** 1 School of Engineering and Science, Tecnologico de Monterrey, Avenida Eugenio Garza Sada 2501 Sur, Monterrey 64700, NL, Mexico; 2 Programa de Posgrado en Alimentos del Centro de la República (PROPAC), Research and Graduate Studies in Food Science, School of Chemistry, Universidad Autónoma de Querétaro, Centro Universitario, Cerro de las Campanas S/N, Santiago de Querétaro 76010, QRO, Mexico; 3 Grupo de Química y Funcionalidad de Carbohidratos y Derivados, Instituto de Investigación en Ciencias de la Alimentación, CIAL (CSIC-UAM), CEI (CSIC + UAM), Nicolás Cabrera, 9, Campus de la Universidad Autónoma de Madrid, 28049 Madrid, Spain; 4 Centro de Investigación en Nutrición y Salud Pública, Facultad de Salud Pública y Nutrición, Autonomous University of Nuevo León, San Nicolás de los Garza 66455, NL, Mexico

**Keywords:** bioactive compounds, mango by-products, dietary fiber, phenolic compounds, functional confectionery, fecal fatty acid profile, lipid excretion, characterization

## Abstract

Dietary fiber and phenolic compounds are key bioactives in gastrointestinal and metabolic health; however, their compositional features and metabolic implications have rarely been studied as an integrated system within realistic food matrices. Mango bagasse confectionery previously demonstrated prebiotic potential, and its reformulation with extruded mango peel showed hepatoprotective effects linked to gut microbiota modulation. In this study, mango bagasse and peel confectionery (MBPC) was characterized and its metabolic impact was evaluated in vivo. Wistar rats were fed standard or high-fat diets with or without MBPC supplementation, followed by fecal fatty acid analysis. MBPC exhibited a high dietary fiber content for a confectionery product (25 g total fiber per 100 g), with monomeric profiles indicative of cell wall-derived polysaccharides and pectic components. The fiber fraction showed a low Mw (14.71 ± 0.02 kDa), suggesting a matrix favorable for fiber–phenolic interactions. Phenolic profiling revealed substantial concentrations of free (9.0 mg/mL) and bound (16.7 mg/mL) phenolic compounds. Fecal fatty acid profiles were diet-dependent, with palmitic acid showing the highest relative abundance, followed by stearic, oleic, and linoleic acids, associated with dietary fiber intake. This study elucidates the structural and metabolic relevance of dietary fiber–phenolic interactions within a formulated food matrix.

## 1. Introduction

Dietary fiber and phenolic compounds are widely recognized as bioactive components of interest in gastrointestinal function and metabolic health. Dietary fiber, particularly its soluble fraction, can be fermented by the gut microbiota and has been associated with physiological effects such as enhanced intestinal transit and the sequestration of lipids and bile acids, thereby reducing their intestinal absorption, increasing fecal excretion, and potentially modulating lipid metabolism [1]. Phenolic compounds, on the other hand, exert antioxidant, antimicrobial, and signaling activities along the gastrointestinal tract [2]. Importantly, the fiber matrix can protect phenolic compounds during gastrointestinal transit, allowing them to reach the colon, where they may exert antioxidant activity or serve as substrates for microbial fermentation [3]. Accordingly, several studies indicate that polyphenols bound to fiber largely escape absorption in the upper gastrointestinal tract and reach the colon, where microbial fermentation contributes to their gradual release and conversion into bioactive phenolic metabolites, such as short-chain fatty acids (SCFAs), thereby extending their biological activity beyond the small intestine [4,5].

Both dietary fiber and phenolic compounds frequently coexist within plant-derived matrices and can be obtained from agro-industrial by-products such as mango peel [6,7]. Recent studies have highlighted the incorporation of fruit juice processing by-products into food matrices and their physiological impact. For instance, the addition of 1% apple pomace resulted in the highest levels of short-chain fatty acids, whereas apricot and peach by-products showed a stronger prebiotic effect on *Lactobacillus acidophilus*, pointing to multiple mechanisms through which fiber-rich by-products may contribute to improved health outcomes [7]. Furthermore, interactions among dietary fiber, polyphenols, and digestive enzymes have been summarized, emphasizing conjugation and complex formation between these components, as well as interactions between specific fibers and a wide variety of polyphenols. To adequately assess the nature of these interactions and their associated health outcomes, they must be studied using targeted and matrix-specific approaches [8].

Despite growing recognition of the nutritional relevance of fiber-associated polyphenols, the interactions between dietary fiber and antioxidants remain insufficiently characterized, particularly with respect to the mechanisms allowing for their release and absorption during gastrointestinal digestion. Experimental studies suggest that polyphenol–fiber interactions can delay polyphenol liberation, alter antioxidant capacity, and promote a sustained bioavailability compared to free polyphenols [8,9]. However, the molecular and physiological processes underlying these effects remain poorly defined, particularly in complex plant-derived matrices. Variations in dietary fiber structure and phenolic composition, along with matrix-dependent interactions, complicate the interpretation of fiber–polyphenol interactions and their associated metabolic outcomes [9]. Moreover, the complexity of plant-based food matrices limits the ability to extrapolate results obtained from isolated compounds, thus the importance of studying fiber–polyphenol interactions in real food systems.

Overall dietary fiber intake remains below recommended levels [10]. For this reason, the formulation of novel foods—such as functional confectionery products—that take advantage of agro-industrial by-products as sources of bioactive compounds represents a promising strategy to help increase fiber consumption. In previous studies, Herrera-Cazares et al. reported the formulation and compositional characterization of a confectionery product formulated with extruded mango bagasse [11], as well as its in vitro fermentability [12] and digestibility [13]. Subsequently, Luzardo-Ocampo et al. demonstrated its in vitro prebiotic effect [14] and conducted a sensory evaluation [15]. More recently, a reformulation of the confectionery was developed through the incorporation of extruded mango peel. This new formulation, composed of MBPC, was evaluated in vivo under obesogenic conditions, where it was shown to exert a potential protective effect against hepatic fat accumulation through modulation of the gut microbiota, characterized by a reduction in the RA of obesogenic bacterial species [16]. In addition, hepatic transcriptomic analysis provided insights into the potential molecular pathways involved in this effect [17]. Therefore, considering the complexity and specificity of health outcomes arising from fiber–polyphenol interactions, the hypothesis of this study is that MBPC contains substantial amounts of dietary fiber and phenolic compounds which, acting synergistically, exert a beneficial effect on gastrointestinal metabolism, particularly by modulating lipid metabolism and secretion. Accordingly, this work aims to elucidate the compositional and structural characteristics of the dietary fiber and phenolic fractions present in MBPC in order to better interpret, through fecal lipid excretion, the metabolic changes previously observed, with particular emphasis on gut microbiota modulation and hepatoprotective effects observed in vivo.

## 2. Results

### 2.1. Dietary Fiber Characterization of MBPC

Proximate analyses and the determination of reducing sugars of MBPC were performed in a previous study and are shown in Table 1. These results indicate that carbohydrates are the main component of MBPC on a dry basis. Dietary fiber represents the major carbohydrate fraction, comprising 14% insoluble fiber and 11% soluble fiber of the total MBPC. In addition to these carbohydrates, fructose and sucrose were the main reducing sugars present in MBPC, accounting for 5% and 4%, respectively.

#### Monomeric Composition of H–MBPC and NH–MBPC and Molecular Weight of MBPC

**Figure 1:**
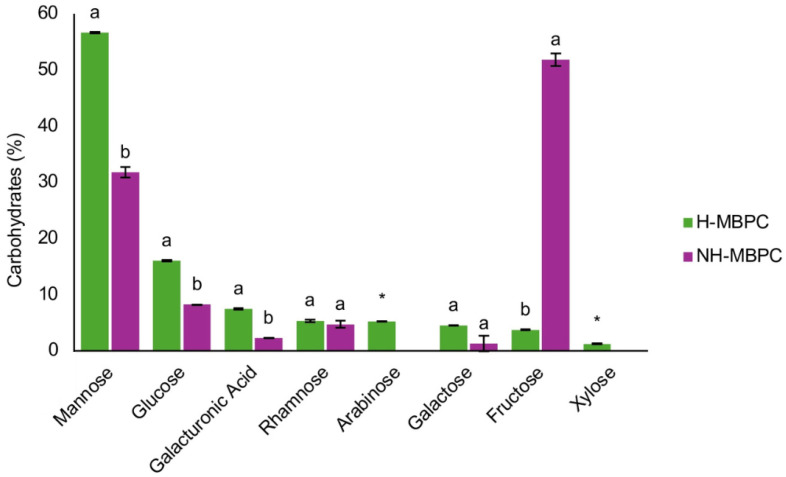
Monomeric composition of H–MBPC and NH–MBPC. Results are expressed as a percentage (%) of total carbohydrates and represent the mean of two independent experiments. Different letters within the same monosaccharide indicate significant differences between treatments (Tukey–Kramer post hoc test, α = 0.05). An asterisk (*) indicates that the monosaccharide was absent in one treatment. All results are expressed on a dry basis.

In order to further characterize the dietary fiber fraction of MBPC, the monomeric composition of H–MBPC and NH–MBPC was analyzed (Figure 1). Significant differences were observed between H–MBPC and NH–MBPC. Mannose was the predominant monomer, accounting for 56.64% in H–MBPC and 31.77% in NH–MBPC, followed by glucose (16.02% in H–MBPC and 8.22% in NH–MBPC). Galacturonic acid was also detected at 7.42% in H–MBPC and 2.25% in NH–MBPC. Rhamnose was present at 5.32% in H–MBPC and 4.71% in NH–MBPC, with no significant differences between treatments. Galactose was detected at 4.46% in H–MBPC and 1.27% in NH–MBPC, also without significant differences. Fructose accounted for 3.73% in H–MBPC and 51.78% in NH–MBPC. Finally, arabinose (5.23%) and xylose (1.19%) were detected exclusively in H–MBPC.

The Mw of the aqueous extract of MBPC, which is assumed to correspond to the insoluble fiber, was estimated at 14.71 ± 0.02 kDa based on duplicate measurements.

### 2.2. Phenolic Compounds of MBPC

The phenolic compound profile of the MBPC is presented in Table 2 and Table 3 corresponding to the fractions of free phenols and bound phenols, respectively. In the free fraction (Table 2), gallic acid, mangiferin and ferulic acid were identified and quantified, with concentrations of 0.58, 3.20 and 5.25 mg/mL, respectively. Among these compounds, ferulic acid had the highest concentration, followed by mangiferin.

In the fraction of bound phenolic compounds (Table 3), a broader profile was observed, with the presence of gallic acid, catechin, mangiferin and quercetin. Catechin was the compound with the highest concentration (9.21 mg/mL), followed by mangiferin (4.72 mg/mL), gallic acid (1.04 mg/mL) and quercetin (1.74 mg/mL).

### 2.3. Fecal Fatty Acid Profile

**Figure 2:**
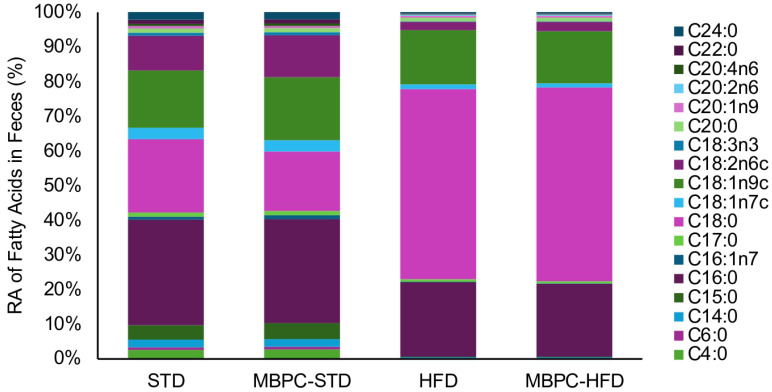
Relative abundance (%) of total fecal fatty acids in experimental dietary groups.

The HFD groups exhibited higher lipid levels in fecal matter than the STD groups (*p* < 0.05), ranging from 160.1 to 220.2 mg FAME/g of feces, compared with 17.7 to 26.2 mg FAME/g of feces. The MBPC–supplemented groups showed a lower average lipid content than their respective control groups; however, these differences were not statistically significant (*p* > 0.05). A total of 45 lipid compounds, ranging from C4 to C24, were screened, of which 18 were detected. The RAs of these compounds are shown in Figure 2. Among them, the fatty acids with the highest RA were C16:0 (palmitic acid), C18:0 (stearic acid), C18:1n9c (oleic acid), and C18:2n6c (linoleic acid).

**Figure 3:**
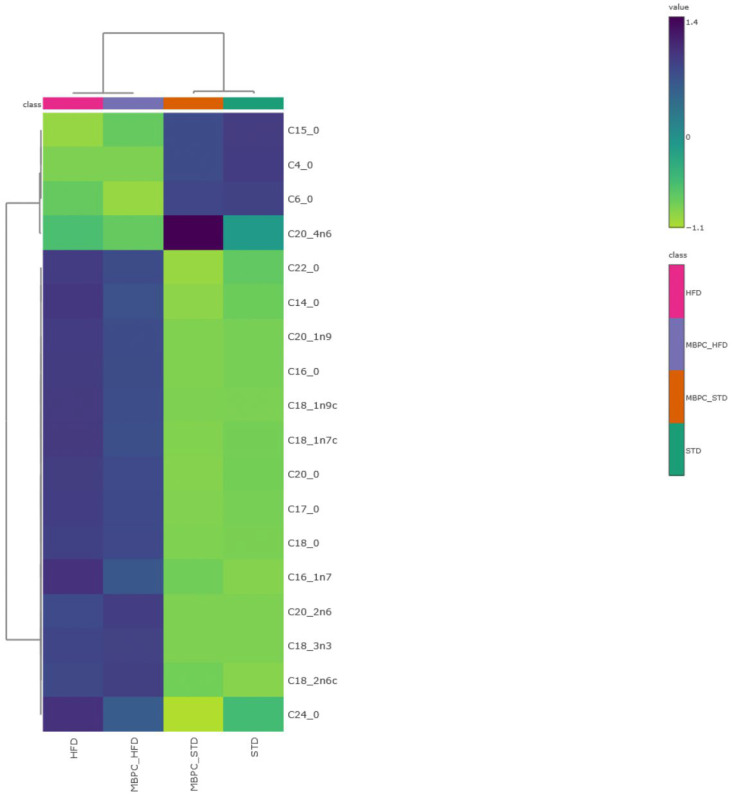
Multivariate analysis of fatty acids from fecal contents. Hierarchical clustering heatmap generated from autoscaled data using Ward’s linkage method and Pearson correlation as the distance measure. Dark blue indicates values above the mean, whereas yellow indicates values below the mean, highlighting similarities among dietary treatments and fatty acid profiles.

To further explore group-level differences in fatty acid profiles, a hierarchical clustering heatmap was generated (Figure 3). This multivariate analysis revealed a clear separation of fatty acid associations according to dietary treatment. The STD groups clustered together and showed stronger associations with fatty acids containing shorter carbon chain lengths, whereas the HFD groups exhibited stronger associations with long-chain fatty acids (C16–C24). Notably, C20:4n6 (arachidonic acid) showed the strongest association with the MBPC–STD group. The heatmap further indicated that long-chain fatty acids—mainly palmitic, stearic, oleic, and linoleic acids—displayed higher color intensity in the MBPC–HFD group compared with the HFD group, suggesting a higher RA of these compounds in feces. This pattern reflects increased fecal lipid excretion in the supplemented animals, whereas the lower intensity observed in the HFD group suggests greater intestinal lipid absorption. Similarly, the STD–groups showed intermediate or low values for these fatty acids, maintaining a more homogeneous fecal lipid profile.

Furthermore, Spearman’s correlation analysis indicated that most fatty acids were positively associated with total dietary fiber intake. The strongest positive correlations were observed for butyric (C4:0, ρ = 0.81), caproic (C6:0, ρ = 0.79), pentadecanoic (C15:0, ρ = 0.80), behenic (C22:0, ρ = 0.82), and lignoceric (C24:0, ρ = 0.82) acids. High positive correlations were also found for myristic (C14:0, ρ = 0.74), palmitic (C16:0, ρ = 0.76), heptadecanoic (C17:0, ρ = 0.78), linoleic (C18:2n6c, ρ = 0.72), α-linolenic (C18:3n3, ρ = 0.73), and arachidonic (C20:4n6, ρ = 0.73) acids. Monounsaturated fatty acids showed moderate correlations, including palmitoleic (C16:1n7, ρ = 0.64), cis-vaccenic (C18:1n7c, ρ = 0.73), oleic (C18:1n9c, ρ = 0.40), eicosenoic (C20:1n9, ρ = 0.57), and eicosanoic (C20:0, ρ = 0.62). In contrast, negative correlations were observed for stearic acid (C18:0, ρ = −0.65) and eicosadienoic acid (C20:2n6, ρ = −0.79).

## 3. Discussion

MBPC had higher fiber content than other products currently available on the market, whose main ingredients—such as water, sucrose, and corn syrup—do not provide dietary fiber [18]. Moreover, the contributions of insoluble and soluble fiber are nearly equivalent (14% and 11%, respectively), a feature more commonly observed in fruits consumed with their peel, such as apples, pears, and berries [19], but not in fruits such as mango, in which soluble fiber predominates in the pulp while insoluble fiber is typically discarded with the peel [6,7,20]. The proximate composition of MBPC is consistent with previous mango bagasse–based functional confectionery formulations reported in the literature. Similar products developed using mango bagasse and peel as fiber sources have shown high dietary fiber content, low lipid levels, and carbohydrates largely derived from structural polysaccharides rather than digestible sugars [11,12,13]. The fiber composition of MBPC results from the integration of extruded fiber derived from both the mango peel and mango bagasse [16]. The consumption of insoluble and soluble dietary fiber has been associated with multiple health benefits, particularly related to gastrointestinal health and lipid metabolism [21], through mechanisms including improved intestinal transit [22], the formation of viscous gels that trap lipids and bile acids and promote their excretion in feces [1,23,24], and modulation of the gut microbiota by reducing obesogenic species and increasing the production of SCFAs that regulate lipid metabolism [16,17,25,26].

The monosaccharides detected in MBPC are characteristic of plant cell wall polysaccharides. Glucose is primarily associated with cellulose, whereas xylose, mannose, arabinose, and galactose are typical components of hemicellulose. Galacturonic acid and rhamnose, together with arabinose and galactose, are characteristic of pectic substances, while fructose is mainly considered a non-structural sugar [27]. Overall, a higher proportion of monosaccharides was observed in H–MBPC, likely due to the hydrolysis of glycosidic bonds during sample processing, resulting in polysaccharide depolymerization and the release of monomeric units. Consequently, monosaccharides previously bound within complex polymeric structures are quantified in their free form, resulting in an apparent increase in their concentration [28]. In contrast, fructose was detected in higher proportions in the NH–MBPC samples, as it is predominantly present in free form or in easily extractable compounds rather than being structurally associated with polysaccharides. In addition, fructose is susceptible to degradation under hydrolysis conditions, which may contribute to its lower quantification in hydrolyzed samples [29,30].

The presence of cell wall-derived monosaccharides, including glucose, xylose, mannose, arabinose, galactose, galacturonic acid, and rhamnose, indicates that MBPC contains carbohydrate fractions associated with dietary fiber functionality, beyond readily digestible sugars such as fructose and sucrose identified in the proximate analysis. From a functional perspective, this carbohydrate profile reflects the presence of hemicelluloses and pectins, which contribute soluble dietary fiber, prebiotic potential, and technological functionalities, including gelation, water retention, and texture enhancement [31]. Consequently, MBPC exhibits features consistent with a functional product beyond its caloric contribution.

Regarding Mw, it is likely that the measured Mw corresponds mainly to the pectic fraction of MBPC. Pectins extracted from Ataulfo mango peel have been reported to exhibit Mw values exceeding 900 kDa, while pectins from other mango varieties may reach up to 2900 kDa [32]. However, MBPC is formulated using extruded mango peel and bagasse, a processing step known to induce fiber fragmentation [11,13], which may explain the substantially lower Mw observed in this study (approximately 14 kDa). This value is nevertheless comparable to those reported for other gelled confectionery products, which typically range from 15 to 4000 kDa [18]. Importantly, low-molecular-weight pectins are capable of interacting with phenolic compounds, forming small and soluble complexes that may facilitate their release in the gastrointestinal tract [11,13,15]. Taken together, the monomeric composition, fiber-associated carbohydrate profile, and reduced molecular weight observed in MBPC are consistent with a plant cell wall matrix in which structural polysaccharides may interact with other bioactive compounds.

In addition to dietary fiber, MBPC exhibits a diverse phenolic profile that contributes to its functional potential, with gallic acid, mangiferin, catechin, ferulic acid, and quercetin identified among the main compounds. These compounds have been consistently reported in mango and its by-products (peel and bagasse), where major phenolic classes, including phenolic acids, flavonoids, and xanthones such as mangiferin, are present in both free and conjugated forms [11,33,34]. In particular, the detection of mangiferin is consistent with previous reports identifying this xanthone as one of the most abundant bioactive compounds in mango bagasse and other by-products of *Mangifera indica* [33].

Furthermore, the presence of flavonoids such as catechin and quercetin in the bound fraction supports previous findings indicating that a substantial proportion of phenolic compounds can be associated with the plant cell matrix and released following hydrolysis [35]. Likewise, phenolic acids such as ferulic and gallic acid have been reported in free fractions of mango pulp and peel [6]. The presence of polyphenols found in fruit by-products as bonded phenolic compounds has been established in advance, these phenolic compounds tend to have strong interactions with the cell wall via non-covalent associations with polysaccharides such as pectin, cells or hemicellulose [36]. These interactions, which tend to be mediated by hydrogen bonds or hydrophobic forces, limit bioaccessibility through the small intestine and allow for the arrival of phenolic compounds to the colon, where it has been reported that they can promote antioxidant and antimicrobial effects [8]. Although the protein content of the formulation is relatively high and proteins are known to interact with polyphenols, previous in vitro studies have shown that the protein fraction—derived mainly from the added gelatin—can act as a protective matrix for phenolic compounds released during the extrusion process [11,13]. In addition, because gelatin is incorporated together with mango peel and bagasse, it is hypothesized that a substantial proportion of phenolic compounds remains primarily associated with dietary fiber, reflecting their natural state of occurrence in fruits. Taken together, these results suggest that MBPC may represent a relevant source of phenolic compounds with functional potential, in agreement with previous studies on mango by-products.

In this context, the phenolic profile obtained in the present study can be compared with that reported for extruded mango bagasse confections (EMBC) by Herrera-Cazares et al. [13], in which phenolic acids and flavonoids associated with the bound fraction predominated, whereas xanthones such as mangiferin were not detected after extrusion. Unlike the EMBC formulation, which was produced exclusively from mango bagasse, the confection evaluated in this study incorporates mango peel into the formulation. Given that mango peel has been identified as a major reservoir of mangiferin [33,37], it is plausible that the fibrous matrix of the peel exerted a protective effect during extrusion, favoring the retention of mangiferin associated with structural components of the matrix and enabling its subsequent release only after the hydrolysis step applied for bound phenolics extraction.

Taking into consideration its combined dietary fiber composition, reduced molecular weight of the fiber matrix, and associated phenolic profile, MBPC exhibits structural and biochemical characteristics that are highly relevant to metabolic responses. Previous studies have shown that polyphenols associated with dietary fiber are not readily released or absorbed in the upper gastrointestinal tract, but instead reach the colon, where microbial fermentation facilitates their gradual liberation and biotransformation into bioactive metabolites [4,36]. This delayed release mechanism has been proposed to extend the temporal window of antioxidant activity and modulate physiological responses through sustained exposure to phenolic metabolites rather than rapid systemic absorption [9,38]. In this context, the reduced molecular weight and altered structural organization of the fiber matrix in MBPC may enhance fermentability and accessibility to the gut microbiota, promoting interactions between fiber-bound phenolics and microbial enzymatic systems [8,9]. Taken together, the structural integration of dietary fiber and phenolic compounds in MBPC supports its potential role as a functional ingredient capable of modulating metabolic responses, reinforcing the importance of studying antioxidant compounds within real food matrices.

The fecal fatty acid profile indicated that lipid excretion was associated with the type of diet consumed, with the STD groups showing greater overall fatty acid excretion. These groups showed a more pronounced association with SCFAs, suggesting that these compounds were produced by the gut microbiota during the fermentation of mango-derived dietary fibers. This observation is consistent with previous studies reporting the presence of butyric acid after in vitro fermentation of mango bagasse confectionery [11,14] and in the cecal contents of in vivo models supplemented with MBPC [17]. During fiber fermentation, polysaccharide structures are broken down, facilitating the release of phenolic compounds that escape absorption in the small intestine and may subsequently act as fermentation substrates, exert selective antimicrobial effects, or contribute antioxidant activity in the colon [39]. In addition, a close association was observed between the MBPC–STD group and arachidonic acid. According to previous studies, this response has been associated with changes in intestinal linoleic acid conversion, which may reflect a lipid metabolic profile compatible with a less pro-inflammatory intestinal environment [40], potentially in relation to increased fiber intake from MBPC.

In contrast, the HFD groups exhibited lower overall fecal lipid excretion; however, the MBPC–HFD group showed higher level of fecal lipid excretion, of fecal lipid excretion, particularly for fatty acids with the highest RA (palmitic, stearic, oleic, and linoleic acids). This pattern is supported by the positive correlations observed between these fatty acids and dietary fiber intake, suggesting that consumption of fiber from MBPC under high-fat conditions may promote fatty acid sequestration and reduce intestinal lipid absorption [1,41]. These associations may reflect changes in lipid handling that are consistent with lower energy uptake and reduced lipid accumulation, in agreement with the reduced hepatic steatosis and adiposity previously reported for rats fed MBPC–HFD [16].

Furthermore, phenolic compounds such as flavonoids present in mango, and consequently in MBPC, have been reported to exert selective antimicrobial effects, particularly against certain Gram-positive gut bacteria [42]. The release of these phenolic compounds in the large intestine may contribute to modulation of the gut microbiota, as reported in previous studies showing reduced relative abundances of obesogenic-associated species—*Ruminococcus gnavus*, *Ruminococcus torques*, *Coprococcus catus*, *Coprococcus comes*, *Clostridium hylemonae*, *Phascolarctobacterium faecium*, and *Blautia* LZLJ-3—in MBPC–supplemented groups [16,17]. Moreover, polyphenols have been described as exerting a dual effect by inhibiting potentially harmful bacteria while supporting the growth of beneficial symbionts [43]. Consistently, MBPC supplementation was associated with higher gut microbiota α-diversity [17], possibly reflecting the combined effects of dietary fiber fermentation and polyphenol availability. Finally, increased fiber intake and higher microbial diversity in MBPC–supplemented groups were negatively correlated with hepatic fat accumulation [16], further supporting the role of gut microbiota modulation.

## 4. Materials and Methods

### 4.1. Characterization of Mango Bagasse and Peel Confectionery

#### 4.1.1. Source and Reported Proximate Analysis of MBPC

The MBPC was formulated and supplied by the Chemistry and Functionality of Carbohydrates Laboratory at Universidad Autónoma de Querétaro (UAQ), Mexico. The formulation included extruded mango bagasse and extruded mango peel as fiber-rich components, mango pulp as the fruit base, and gelatin and pectin as gelling agents, with citric acid added as a preservative, using food-grade ingredients sourced locally. Its proximate composition and reducing sugars content were determined and reported in a previous study [16].

#### 4.1.2. Determination of Dietary Fiber Monomers of MBPC

The protocol proposed by Muñoz-Almagro et al. [44] was followed, with slight modifications, as described in Álvarez-Chávez et al. [45]. The following was performed on the hydrolyzed samples (H–MBPC): 30 mg of the sample were combined with 1.5 mL of 2 M trifluoroacetic acid (TFA) in vials sealed under a nitrogen atmosphere. The sealed vials were heated at 110 °C for 4 h, followed by vacuum evaporation. An internal standard (400 μL of 0.5 mg/mL β-phenyl glucoside) was added, the samples were re-evaporated, and derivatization was carried out for GC-FID analysis, with all procedures performed in duplicate. For the non-hydrolyzed samples (NH–MBPC), the procedure was carried out starting from derivatization.

Analyses were conducted using an Agilent Technologies gas chromatograph (GC7890A; Agilent Technologies, Santa Clara, CA, USA) equipped with a flame ionization detector (FID) and a 7693 automatic injector. Separation of the oxime-derived trimethylsilyl (TMSO) derivatives was achieved using a DB-5HT fused silica capillary column (30 m × 0.32 mm × 0.10 μm; J&W Scientific, Agilent Technologies, Folsom, CA, USA). Nitrogen served as the carrier gas at a flow rate of 1 mL/min. The detector temperature was set at 280 °C, and the oven temperature was programmed to increase from 150 °C to 380 °C at 10 °C/min. Samples were injected in split mode with a 1:20 ratio. Data acquisition and integration were performed using Agilent ChemStation software Rev. E.02 (Agilent Technologies, Santa Clara, CA, USA). All determinations were performed in duplicate. Results are expressed as mean ± standard deviation. Experimental reproducibility was evaluated by calculating the relative standard deviation (RSD, %) for each monosaccharide. RSD values below 5% were considered indicative of good analytical reproducibility, while higher values were attributed to low-abundance compounds or concentrations close to the detection limit.

#### 4.1.3. Estimation of the Molecular Weight of Dietary Fiber of MBPC

The molecular weight (Mw) estimation followed Ferreira-Lazarte’s et al. [46]. protocol, involving the preparation of a sample solution of 5 mg/mL in water, subsequent dilution to 1 mg/mL and filtration through a 0.45 μm pore size filter. The filtered sample was placed in an HPSEC-ELSD vial. Analysis utilized an Agilent Technologies LC 1260 Infinity (Agilent Technologies, Santa Clara, CA, USA) system with TSK-GEL Columns (Tosoh Bioscience, Tokyo, Japan), a TSK-Gel precolumn, a mobile phase of 0.1 M ammonium acetate, a flow rate of 0.5 mL/min at 30 °C, and a 20 μL sample injection. An evaporative light scattering detector (ELSD) at 30 °C and external calibration with pullulan patterns were employed for Mw determination. The calibration curve had the elution volume on the x-axis and the logarithm of Mw on the y-axis, using known Mw values (805, 200, 10, 3, and 0.3 kDa) of commercial pululan patterns (Fluka Analytical, Buchs, Switzerland)) at a concentration of 1 mg/mL.

#### 4.1.4. Extraction and Identification of Phenolic Compounds of MBPC

Free and bound phenolics compounds were extracted according to the methodology described by Herrera-Cazares et al. [13]. Briefly, free phenolics were obtained by stirring 1 g of MBPC in an ethanol/water mixture (80:20, *v*/*v*), followed by centrifugation. The supernatant was concentrated under vacuum and resuspended in 1 mL of HPLC-grade methanol. The remaining pellet was subjected to alkaline hydrolysis with NaOH (2 M), neutralized with HCl, defatted with hexane, and extracted with ethyl acetate. The extract was concentrated and resuspended in 1 mL of HPLC-grade methanol. Both samples were stored at −20 °C until analysis.

The determination of the phenolic profile was performed as follows. Briefly, the extract obtained from both bound phenolic compounds and free phenolic compounds was filtered using a 0.45 μm membrane in order to remove suspended solids. A chromatographic analysis was performed using an Agilent 1100 Series HPLC system (Agilent Technologies, Palo Alto, CA, USA) equipped with a diode array detector (DAD) for multi-wavelength detection. A Zorbax Eclipse XDB-C18 column (Agilent Technologies, Santa Clara, CA, USA; 4.6 × 250 mm, 5 μm particle size) under reversed-phase conditions was used for compound separation. The mobile phase was composed of a solution of water to which 1% acetic acid (*v*/*v*) and acetonitrile was added to which 1% acetic acid (*v*/*v*) was added, the conditions followed the protocol reported by Ramírez-Jiménez et al. [47] with some modifications. The flow rate was maintained at 0.75 mL/min, and the temperature of the column was set at 40 °C. Chromatographic separation was carried out using a gradient elution composed of water and acetonitrile. The mobile phase initially contained 5% acetonitrile at 0 min, which was increased to 15% at 5 min, 25% at 7 min, 30% at 10 min, 35% at 12 min, 40% at 15 min, 50% at 20 min, 90% at 25 min, and 95% at 30 min. Subsequently, the acetonitrile content was reduced to 50% at 31 min and returned to 5% at 32 min to allow column re-equilibration prior to the next injection. The gradient was applied under constant flow conditions. Detection was performed at 280 nm for phenolic acids and 320 for flavonoids, while full spectral data (190–600 nm) were recorded for compound identification. Quantification was done using calibration curves with the following concentrations: 0.01, 0.025, 0.050, 0.075, 0.1 mg/mL, prepared with standards of mangiferin, gallic acid, catechin, ferulic acid and quercetin. Individual compounds were identified by comparing retention times and UV-Vis spectra with those of the reference standards.

### 4.2. In Vivo Experimental Design and Fecal Lipid Analysis

#### 4.2.1. Animals, Experimental Design, and Dietary Supplementation

An in vivo experimental procedure was carried out in Wistar rats. The protocol was approved by the Ethical Committee of UAQ (approval ID: CBQ21/015, date 24 May 2021), and the procedures followed the Mexican standard NOM-062-ZOO-1999 [48] and the National Institutes of Health (NIH) Guidelines for the Care and Use of Laboratory Animals. As described in our latest publication [17], twenty-four male Wistar rats (4 weeks old, average weight 119 g) were randomly assigned to four groups (*n* = 6, based on reports from previous similar studies [49,50]): standard diet (STD), standard diet supplemented with MBPC (MBPC–STD), high-fat diet (HFD; 60% energy from fat), and high-fat diet supplemented with MBPC (MBPC–HFD). Animals were housed individually under controlled environmental conditions (temperature, 22 ± 4 °C; relative humidity, 50 ± 15%; 12 h light/dark cycle), and the supplementation was administered daily and adjusted to provide 5.9 g of fiber per 1000 kcal consumed, in accordance with the daily intake recommendations and Mexican children’s average consumption of dietary fiber [51,52] for 11 consecutive weeks according to similar experimental studies [53,54,55].

#### 4.2.2. Fecal Lipid Extraction and Fatty Acid Analysis

After 11 weeks of supplementation with MBPC, feces from cages were collected in Falcon tubes and stored at −80 °C until use. Fecal lipids were identified and quantified by GC-FID (Agilent 7820A, Agilent Technologies, Santa Clara, CA, USA) with EZChrom Elite software (Version 3.3.2.) control and a GC-26 column (Agilent DB-23, Agilent Technologies, Santa Clara, CA, USA; Ref 122-2362; 250 °C; 60 m × 250 µm × 0.25 µm). Previously, feces were lyophilized and derivatized according to the method of Lee et al. [56]. A volume of 1 µL of the sample was injected with a split ratio of 40:1, and the injector temperature was 250 °C. The run consisted of a 1 min start at 50 °C, a ramp of 25 °C/min up to 175 °C, and another ramp of 4 °C/min up to 230 °C. The carrier gas was helium with a flow of 1 mL/min. The detector was maintained at 260 °C, with an H_2_ flow of 40 mL/min. For the identification of fatty acids, the reference material Supelco 37 Component FAME Mix (Sigma-Aldrich, St. Louis, MO, USA) was used, and for quantification, C13:0 was used as an internal standard.

### 4.3. Statistical Analysis

For the determination of monomers, Mw, and phenolic compounds, all measurements were performed in duplicate. The percentage of the monomeric composition of H–MBPC and NH–MBPC was calculated and compared using one-way analysis of variance (ANOVA) followed by a Tukey–Kramer post hoc test, with a significance level of α = 0.05. Prior to parametric analyses, data were evaluated for normality using the Shapiro–Wilk test and for homogeneity of variances using Levene’s test. Mw and phenolic compounds are reported as the mean of the measurements and their corresponding standard deviation. Statistical analyses were performed using Minitab Statistical Software (Minitab 22.2.1, LLC, 2021, State College, PA, USA).

Fatty acid data were averaged within each experimental group (*n* = 6), and relative abundances (RA) were subsequently calculated. Differences in fatty acid abundance among groups were assessed by one-way analysis of variance (ANOVA) followed by a Tukey–Kramer post hoc test (α = 0.05). Multivariate analyses were performed using MetaboAnalyst 6.0 (www.metaboanalyst.ca, accessed on 28 December 2025). Data were normalized by autoscaling, and clustering was conducted using Ward’s method. Correlation analysis between total dietary fiber intake and individual fatty acids was carried out using Spearman’s rank correlation coefficient in MetaboAnalyst 6.0 (www.metaboanalyst.ca, accessed on 28 December 2025).

## 5. Conclusions

In this study, the dietary fiber and phenolic composition present in a confection formulated with extruded mango bagasse and peel was characterized to provide a compositional framework for understanding its potential metabolic relevance. MBPC exhibited a high dietary fiber content (25%), with a balanced distribution of insoluble and soluble fractions. Monomeric profiling indicated that the fiber fraction is mainly composed of cell wall-derived polysaccharides from the peel and pectic components originating from the bagasse, while molecular weight analysis revealed a relatively low Mw (~14 kDa), likely associated with extrusion processing. This combination of compositional features is consistent with a fiber matrix capable of interacting with phenolic compounds and influencing their behavior along the gastrointestinal tract. Although the results demonstrate the presence of dietary fiber, phenolic compounds, and antioxidant capacity, the evidence presented does not fully meet all the criteria proposed by Saura-Calixto for the explicit classification of dietary fiber as antioxidant fiber. Nevertheless, the compositional characteristics observed are promising and support further investigation into fiber–polyphenol interactions in complex food matrices.

The associated phenolic profile, including gallic acid, mangiferin, catechin, ferulic acid, and quercetin, supports MBPC as a source of coexisting dietary fiber and polyphenols within a formulated food. Although the fecal lipid profile analysis was exploratory, the observed associations between dietary fiber intake and lipid excretion are consistent with enhanced fermentability and lipid sequestration, providing a plausible link between fiber composition and previously reported metabolic outcomes. This study presents some limitations, including the use of an animal model, which may restrict the direct translatability of the results to humans. Additionally, the relatively small sample size and short intervention period may limit the detection of long-term effects. Overall, these findings emphasize the importance of compositional characterization of bioactive components within real food matrices to better interpret their interactions with the gut environment and their potential contribution to metabolic modulation, laying the groundwork for future studies focused on causal mechanisms.

## Figures and Tables

**Table 1:** Composition of Mango Bagasse and Peel Confectionery.

Component	Quantity (Per 100 g of MBPC)
Moisture	48 g
Protein	10 g
Lipids	<0.1 g
Ash	0.8 g
Carbohydrates	41 g
Soluble fiber	11 g
Insoluble fiber	14 g
Fructose	5 g
Sucrose	4 g

Proximate analysis was performed on a dry basis, and all values were calculated and reported per 100 g of MBPC.

**Table 2:** Free phenolic compound profile of the analyzed sample determined by HPLC. Concentrations are expressed as mg/mL and reported as mean ± standard deviation (SD) of replicate injections.

Compound	λ (nm)	Concentration (mg/mL)	SD
Gallic Acid	280	0.58	0.06
Mangiferin	360	3.2	0.11
Ferulic Acid	280	5.25	0.25

**Table 3:** Bond phenolics compound profile of the analyzed sample determined by HPLC. Concentrations are expressed as mg/mL and reported as mean ± standard deviation (SD) of replicate injections.

Compound	λ (nm)	Concentration (mg/mL)	SD
Gallic Acid	280	1.04	0.11
Catechin	360	9.21	0.87
Mangiferin	360	4.72	0.2
Quercetin	360	1.74	0.88

## Data Availability

The data supporting the findings of this study are available from the corresponding author upon reasonable request.

## Abbreviations

The following abbreviations are used in this manuscript:

MBPC	Mango bagasse and peel confectionery
RA	Relative abundance
UAQ	Universidad Autónoma de Querétaro
H–MBPC	Hydrolyzed mango bagasse and peel confectionery
TFA	Trifluoroacetic acid
NH–MBPC	Non-hydrolyzed mango bagasse and peel confectionery
TMSO	Oxime-derived trimethylsilyl
Mw	Molecular weight
NIH	National Institute of Health
STD	Standard diet
MBPC–STD	Standard diet supplemented with mango bagasse and peel confectionery
HFD	High-fat diet
MBPC–HFD	High-fat diet supplemented with mango bagasse and peel confectionery
SCFA	Short-chain fatty acids
EMBC	Extruded mango bagasse confections
